# (±)-2-Cyclohexyl-5-methoxy-2H-chromene, a Synthetic 5‑Methoxyflavone Derivative, Is a Selective DNA Polymerase‑β Inhibitor with Neuroprotective Activity against β‑Amyloid Toxicity

**DOI:** 10.1021/acschemneuro.5c00712

**Published:** 2025-12-10

**Authors:** Salvatore Guccione, Sara Merlo, Silvia Tagliapietra, Matteo Pappalardo, Arianna Binello, Alessandro Barge, Livia Basile, Maria Angela Sortino, Giancarlo Cravotto, Agata Copani

**Affiliations:** † Department of Drug and Health Sciences, University of Catania, 95125 Catania, Italy; ‡ Department of Drug Science and Technology, University of Torino, 10125 Torino, Italy; § Department of Biomedical and Biotechnological Sciences, University of Catania, 95123 Catania, Italy

**Keywords:** neuronal apoptosis, neuronal cell cycle reactivation, neuroprotection, DNA polymerase-ß, ß-amyloid

## Abstract

DNA polymerase-β (DNA pol-β) plays a critical role in β-amyloid-induced neurodegeneration by mediating aberrant DNA replication in postmitotic neurons. In previous work, we demonstrated that 5-methoxyflavone inhibits DNA pol-β, though computational analyses suggested potential binding to the primase p58 subunit. Through molecular modeling, here, we designed (*S*)-2-cyclohexyl-5-methoxy-2*H*-chromene (*S*-chromene), a novel flavone-derived inhibitor exhibiting strong electrostatic complementarity with DNA pol-β but weak interaction with primase p58, suggesting enhanced selectivity. (*R*)-2-cyclohexyl-5-methoxy-2*H*-chromene (*R*-chromene) exhibited indistinguishable binding properties from *S*-chromene. The compound was obtained as a racemic mixture (chromene). Since the separated enantiomers were unstable, all biological assays used the racemate. DNA polymerase activity assay confirmed that chromene inhibited selectively DNA pol-β without affecting the primase/DNA pol-α complex activity. Also, the compound amplified methylmethanesulfonate toxicity in wild-type but not DNA pol-β-null fibroblasts, validating target-engagement. In cultured neurons, chromene effectively prevented β-amyloid-induced DNA replication and apoptosis. Ours is the first demonstration of a chromene acting as a selective DNA pol-β inhibitor endowed with a unique mechanism of neuroprotection.

## Graphical abstract

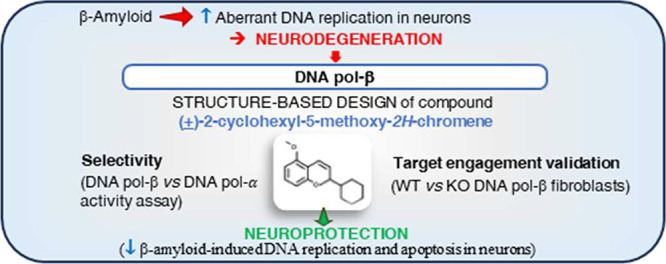

## 1 Introduction

Alzheimer’s disease (AD) is a neurodegenerative pathology representing the leading cause of dementia in the Western world. The characterizing clinical manifestation is a progressive cognitive impairment, which is severely disabling in advanced stages. The pathogenesis of AD is strongly linked to the accumulation of amyloid precursor protein (APP)-derived amyloid-beta (Aβ) peptides, particularly the Aβ_(1–42)_ isoform, which aggregates into soluble oligomers and insoluble plaques, triggering synaptic failure and neuronal apoptosis.^1^ Over the past decade, therapeutic strategies targeting Aβ clearance have been explored, including the development of monoclonal antibodies such as aducanumab, lecanemab, and donanemab, which selectively bind and promote the removal of Aβ aggregates.^2^ While these immunotherapies have shown promise in reducing amyloid burden, their clinical benefits remain modest,^3^ underscoring the need for complementary approaches that address downstream neurodegenerative mechanisms.

A key contributor to AD progression is the aberrant re-entry of postmitotic neurons into the cell cycle, leading to incomplete DNA replication and neuronal death —a process also implicated in other neurodegenerative diseases like Parkinson’s disease and amyotrophic lateral sclerosis.^4,5^ Central to this mechanism is DNA polymerase-β (DNA pol-β), a repair enzyme that mediates a noncanonical DNA replication process in neurons upon Aβ exposure.^6,7^ Given its role, selective inhibition of DNA pol-β presents a promising therapeutic strategy, as it may prevent neuronal cell-cycle activation without affecting proliferating cells.

In previous work, we identified 5-methoxyflavone as a natural-derived DNA pol-β inhibitor capable of blocking Aβ-induced neurodegeneration.^8^ 5-Methoxyflavone belongs to the flavonoid family, polyphenolic plant metabolites exhibiting broad biological activities such as antioxidant, anti-inflammatory, and anticancer effects. These actions are primarily mediated through the direct scavenging of free radicals, cyclooxygenase inhibition, and modulation of key signaling pathways like PI-3K/AKT, ERK1/2, and NF-κB.^9^ Distinctly, 5-methoxyflavone is noted for its anxiolytic properties, which involve interactions with GABA_A_ and 5-HT1A receptors,^10^ and is uniquely characterized by its inhibition of DNA pol-β.^8^ 5-Methoxyflavone binds to the DNA pol-β lyase domain. Because this domain is structurally similar to the p58 subunit of the core replicative polymerase complex (primase/polymerase-α),^11^ further optimization is required to enhance the compound’s selectivity. Here, we present the synthetic derivative of 5-methoxyflavone, (±)-2-cyclohexyl-5-methoxy-2*H*-chromene, which retains its neuroprotective properties while exhibiting improved specificity for DNA pol-β over the primase complex, offering a refined approach for targeting neuronal cell-cycle dysfunction and death.

## 2 Results and Discussion

### 2.1 Molecular Modeling

In previous work, we demonstrated that 5-methoxyflavone inhibits DNA pol-β. However, computational analyses suggested potential binding to the primase p58 subunit of the primase/DNA pol-α complex.^11^ To develop more selective DNA pol-β inhibitors, we employed Cresset’s Spark software (v10.5.5) to systematically explore novel scaffold variants derived from 5-methoxyflavone (https://cresset-group.com/software/spark-databases-current).^12^ The computational workflow consisted of two principal phases: scaffold generation and subsequent evaluation using field-based similarity metrics. This process yielded 16 distinct molecular scaffolds (designated cmd1 through cmd16), each representing a unique structural variation on the parent flavone core (Figure S1). Evaluation of the generated scaffolds was conducted using Cresset’s field-based similarity approach, which provides a comprehensive assessment of molecular complementarity through three primary metrics: complementarity (i.e., a holistic measure of molecular similarity incorporating both electrostatic and steric components); complementarity *R* (i.e., a specialized metric focusing on electrostatic field alignment); complementarity Rho (i.e., a pure shape comparison metric independent of electronic properties). The computational screening revealed a spectrum of complementarity scores across the 16 scaffold variants. Two particular candidates, cmd1 and cmd10, achieved the highest scores across all three metrics and demonstrated nearly identical performance. The remarkable similarity in their field-based descriptors suggests these represent stereoisomeric variants of the same core structure (namely, (*R*)-2-cyclohexyl-5-methoxy-2*H*-chromene and (*S*)-2-cyclohexyl-5-methoxy-2*H*-chromene).

Then, we assessed the electrostatic complementarity of DNA pol-β and the primase p58 subunit with (*S*)-2-cyclohexyl-5-methoxy-2*H*-chromene (*S*-chromene) and (*R*)-2-cyclohexyl-5-methoxy-2*H*-chromene (*R*-chromene), in comparison with 5-methoxyflavone, using Flare, version V10.0.1, (www.cresset-group.com/flare),^11–13^ a software employing the XED force field electrostatic interactions that are fundamental to molecular recognition and stabilization of protein–ligand complexes. Poses of both *S* and *R* enantiomers of chromene were evaluated for essential interactions.

The concept of electrostatic complementarity — the degree to which the electrostatic potentials of interacting surfaces align in a favorable, oppositely charged manner — has become a key consideration in modern drug design. Flare, developed by Cresset, is a comprehensive drug design platform that allows for detailed analysis of electrostatic complementarity. Unlike traditional molecular docking methods, which mainly focus on steric fit and hydrogen bonding, Flare uses field-based approaches to map electrostatic potentials and quantify their complementarity with the binding site. Within Flare, users can calculate and visualize the electrostatic surfaces of both the ligand and the protein. The electrostatic complementarity score (EC score) measures how well the ligand’s electrostatic features align with those of the protein binding site. This helps identify regions of electrostatic mismatch, guiding targeted chemical modifications to improve binding affinity and selectivity.^12^ For easy interpretation, electrostatic potential maps in Flare are color-coded:

- Red regions: Negative electrostatic compatibility
- Green regions: Positive electrostatic compatibility
- White regions: Near-zero electrostatic compatibility

Therefore, an electrostatic compatibility analysis was performed to evaluate the interaction profiles of 5-methoxyflavone, *S*-chromene and *R*-chromene with the two human enzymatic targets: DNA pol-β and primase p58 subunit. Preliminary docking studies of the two enantiomers, *S*-chromene and R-chromene, with both DNA pol-β and the primase p58 subunit yielded highly similar results (docking score difference <0.02), along with overlapping electrostatic complementarity maps, as illustrated in Figure 1.

**Figure 1:**
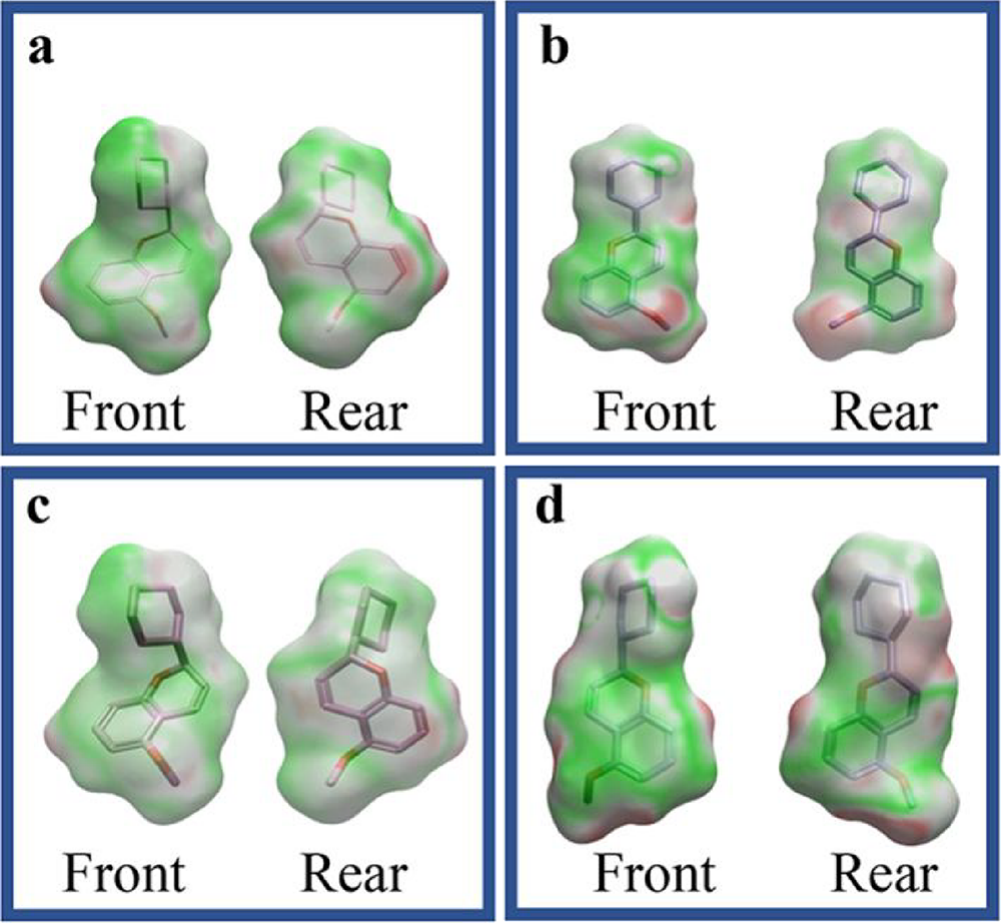
Electrostatic potential surfaces of *S*-chromene interacting with DNA pol-β (a) and the primase p58 subunit (b), along with *R*-chromene interacting with DNA pol-β (c) and the primase p58 subunit (d). The surfaces depict the molecular potential distribution, with green indicating positive potential (favorable interactions), red representing negative potential (unfavorable interactions), and white/gray denoting neutral regions.

Based on these findings, subsequent analyses were conducted using only the *S*-enantiomer.

*S*-chromene exhibited a highly complementary electrostatic distribution with DNA pol-β, featuring extensive regions of favorable potential (Figure 2a), indicative of strong binding affinity. In contrast, its interaction surface with the primase p58 subunit contained more unfavorable electrostatic regions (Figure 2b), suggesting weaker compatibility and, likely, reduced affinity.

**Figure 2:**
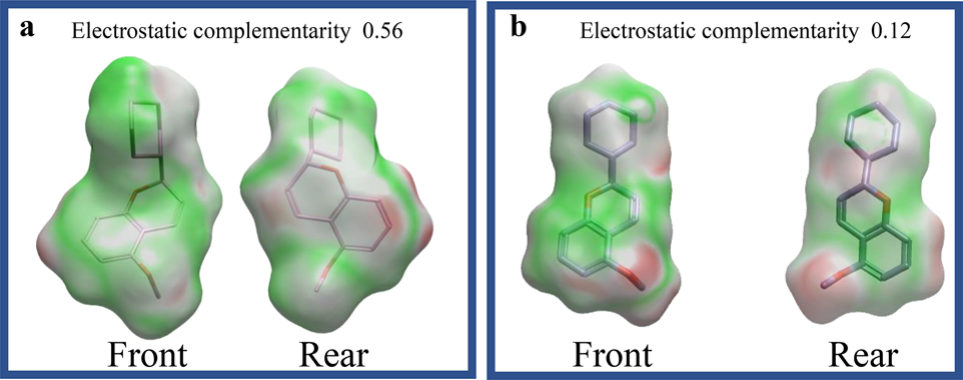
Electrostatic potential surfaces of *S*-chromene interacting with DNA pol-β (a) and the primase p58 subunit (b). The electrostatic surfaces depict the distribution of molecular potentials, where green represents regions of positive potential (favorable for interactions), red indicates negative potential (unfavorable for interactions), and white/gray corresponds to neutral regions. S-chromene exhibits higher electrostatic complementarity with DNA pol-β than with the primase p58 subunit, suggesting greater binding selectivity for the former.

On the contrary, 5-methoxyflavone displayed a more balanced electrostatic profile across both targets (Figure 3a,b). While its compatibility with DNA pol-β was lower than that of *S*-chromene, it retained moderately favorable interactions with both enzymes, implying broader binding potential, but lower selectivity. Collectively, these results suggested that *S*-chromene had a selective and strong binding affinity to DNA pol-β.

**Figure 3:**
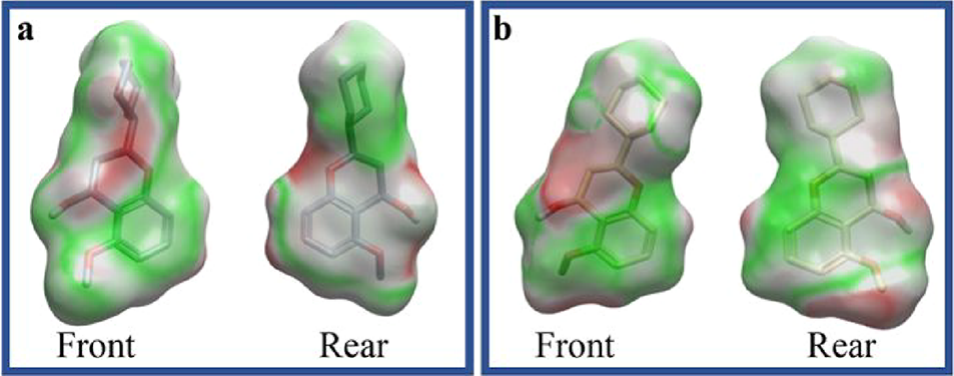
Electrostatic potential surfaces of 5-methoxyflavone in complex with DNA pol-β (a) and the primase p58 subunit (b). The surfaces depict the distribution of molecular electrostatic potentials, where green denotes regions of positive potential (favorable for interactions), red indicates negative potential (unfavorable for interactions), and white/gray represents neutral areas. 5-Methoxyflavone exhibits a relatively balanced interaction profile with both targets, suggesting lower binding selectivity.

The interaction between *S*-chromene and DNA pol-β (PDB ID: 5DQ8) was further analyzed using FLARE’s docking tool (version 10.01). The compound exhibited a strong binding affinity to DNA pol-β, with a docking score of – 5.333, facilitated by hydrophobic interactions with GLU71, GLU75, and ASP74, along with a hydrogen bond with LYS72. In contrast, binding to p58 primase was weaker (docking score: −7.159), involving hydrophobic contacts with ARG306, MET307, TRP327, ILE349, TYR345, and ASN348, as well as a hydrogen bond with ARG306.

For comparison, the docking scores of 5-methoxyflavone were −3.345 for DNA pol-β and −3.896 for the primase p58 subunit. This indicated that 5-methoxyflavone had a high affinity for both DNA pol-β and p58 primase, with a poor selectivity evidenced by the minimal difference in its binding scores for the two enzymes.

Overall, docking scores revealed a clear distinction in the affinity and selectivity profiles of the two compounds. 5-Methoxyflavone exhibited a good affinity for both p58 primase and DNA pol-β, consistent with its small, planar structure facilitating partial accommodation within the active sites of each enzyme. In contrast, *S*-chromene demonstrated significantly higher affinity for DNA pol-β. This enhanced binding was characterized by a greater number of specific hydrophobic interactions within the deeper binding pocket of DNA pol-β. Conversely, the increased steric bulk and altered electronic properties of *S*-chromene resulted in poor complementarity with the primase active site, leading to a markedly lower docking score. This differential binding efficacy rationalizes the *S*-chromene superior selectivity for DNA pol-β over primase.

### 2.2 Chemistry

The total synthesis of chromene (Figure 4) was performed following reported procedures.^14,15^ The synthesis began with 2,6-dihydroxyacetophenone as the starting material. Following methylation, a base-catalyzed condensation with cyclohexanecarbaldehyde yielded 2-cyclohexyl-5-methoxychroman-4-one.

**Figure 4:**
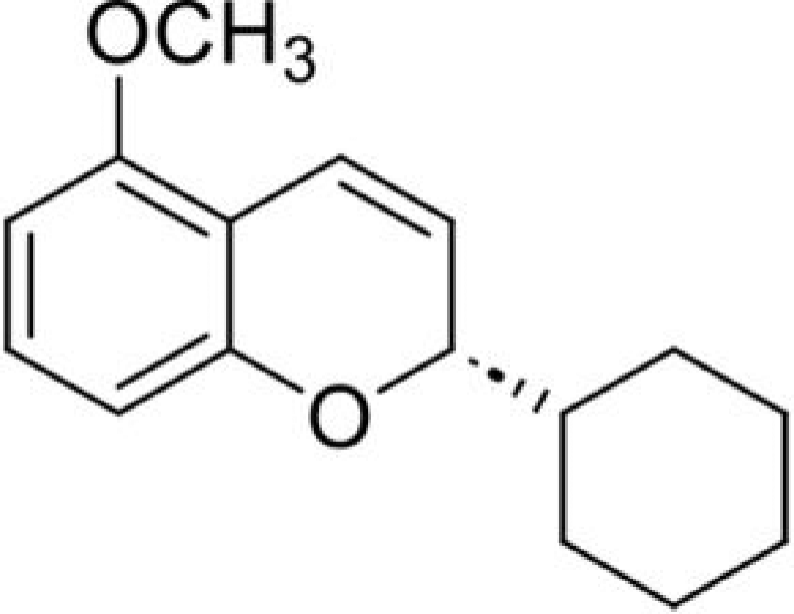
Chemical structure of (*S*)-2-cyclohexyl-5-methoxy-*2H*-chromene.

The target product was then obtained through the reduction of 2-cyclohexyl-5-methoxychroman-4-one to 2-cyclohexyl-5-methoxychroman-4-ol, followed by dehydration (Figure 5). Attempts to achieve asymmetric *S*-chromene transfer hydrogenation of the β-chromanone using ruthenium catalysis^16^ were unsuccessful. Reduction with NaBH_4_ produced 2-cyclohexyl-5-methoxychroman-4-ol as a 3:1 mixture of diastereoisomers. 1D NOE experiments confirmed the *cis* stereochemistry of the major diastereoisomer. Specifically, the most intense NOE correlations were observed between protons H-2 (3.85 ppm), H-3a (2.30 ppm), and H-4 (5.10 ppm), indicating their spatial proximity on the same side of the molecular plane (Figure 6). Irradiation of H-4 showed strong NOE effects with H-2 and H-3a, but not with H-3b (1.95 ppm). Conversely, irradiation of H-2 and H-3a only affected the signals of the other two protons, while H-3b exhibited no significant correlations (Figure 7).

**Figure 5:**
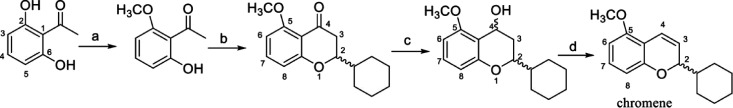
Synthesis pathway of (±)-2-cicloesil-5-metossi-*2H*-cromene (chromene). (a) K_2_CO_3_, Me_2_SO_4_, Me_2_CO, 50 °C; (b) C_6_H_11_CHO, pyrrolidine, MeOH, 50 °C; (c) NaBH_4_, MeOH, 0 °C; and (d) DMSO dry, 145 °C.

**Figure 6:**
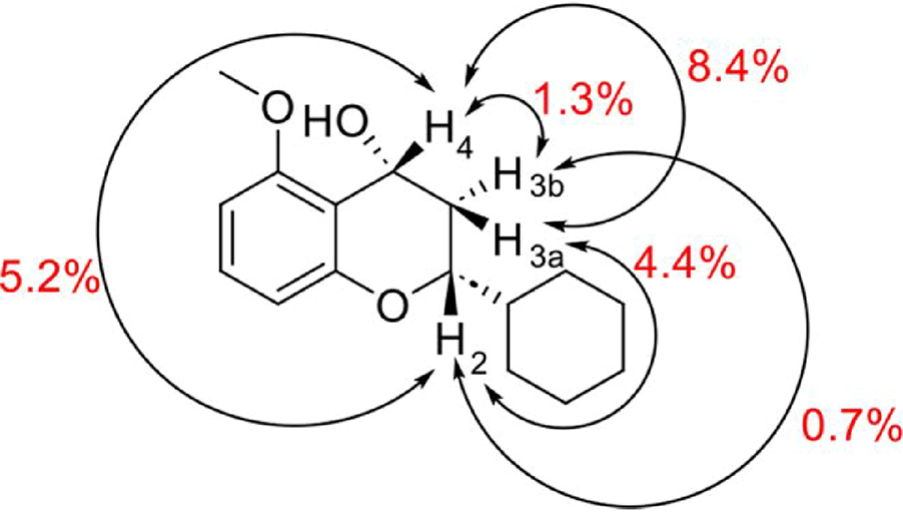
NOE effect (estimated intensity) between the protons 2, 3a, 3b, and 4 in the most abundant *cis* isomer.

**Figure 7:**
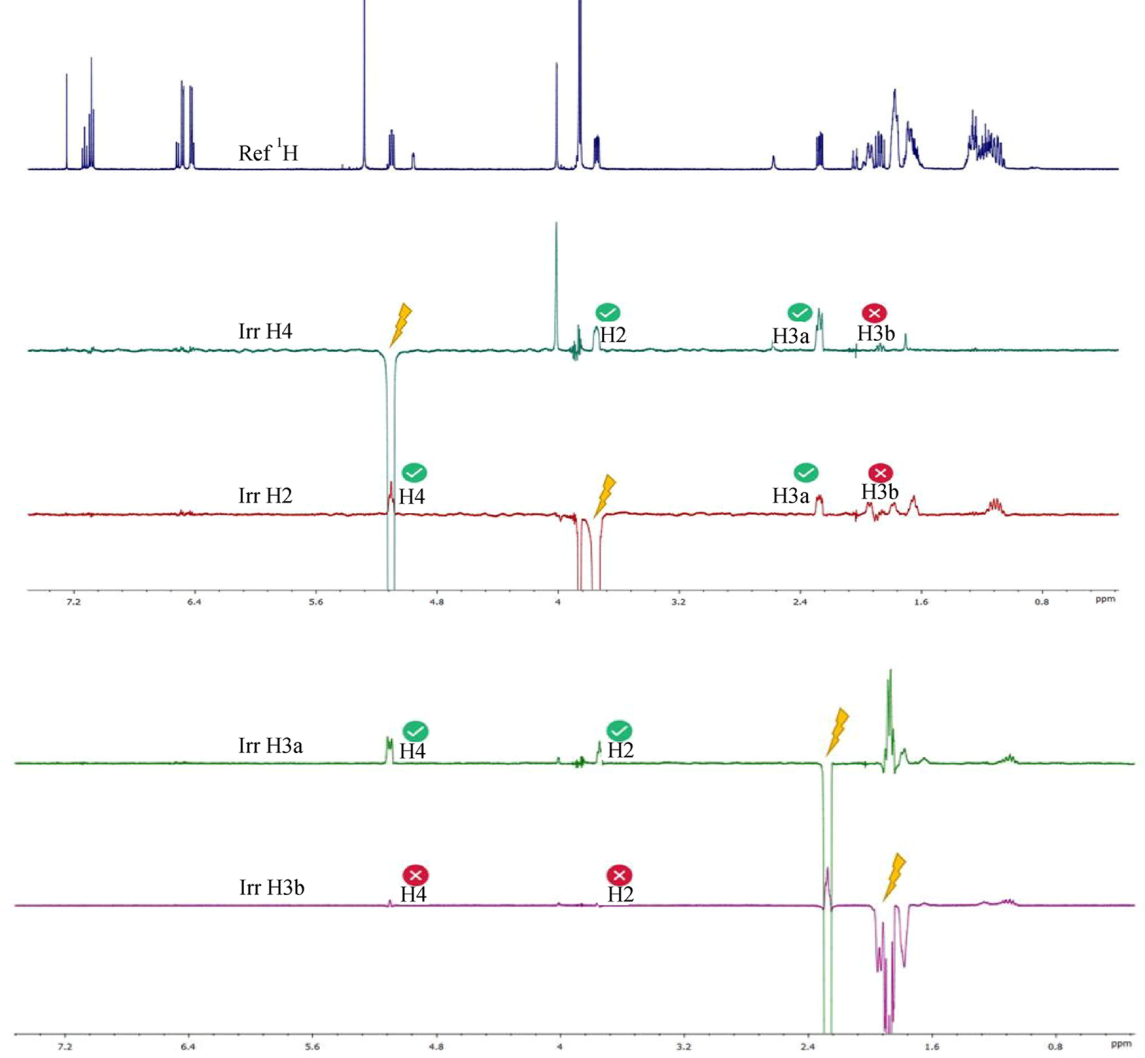
1D NOE spectra. (a) ^1^H NMR standard spectrum; (b) 1D NOE spectrum obtained by irradiating H-4 signal; (c) 1D NOE spectrum obtained by irradiating H-2 signal; (d) 1D NOE spectrum obtained by irradiating H-3a signal; and (e) 1D NOE spectrum obtained by irradiating H-3b signal.

The final dehydration step was performed in dry DMSO^17^ to prevent acid-mediated in situ dimerization of the chromene via double-bond migration (e.g., flav-3-ene isomerizing to flav-2-ene, as reported in ref 18).

Stability studies via ^1^H NMR, conducted weekly over three months on chromene stored at 2 °C, monitored the appearance of diagnostic dimer signals.^19^ The compound remained stable for 2.5 months under these conditions.

Enantiomeric resolution was achieved using HPLC-UV with a semipreparative column having chiral cellulose tris selector, which was chosen based on preliminary trials. However, absolute stereochemical assignment was not possible due to the planar structure of the molecule, which rendered NOE experiments inconclusive, and the inability to obtain suitable crystals for X-ray diffraction analysis. Instead, optical rotation measurements identified the enantiomer with a retention time (r.t.) of 9.45 min as the dextrorotatory form (A), while the peak at 10.80 min corresponded to the laevorotatory antipode (B) (Figure 8).

**Figure 8:**
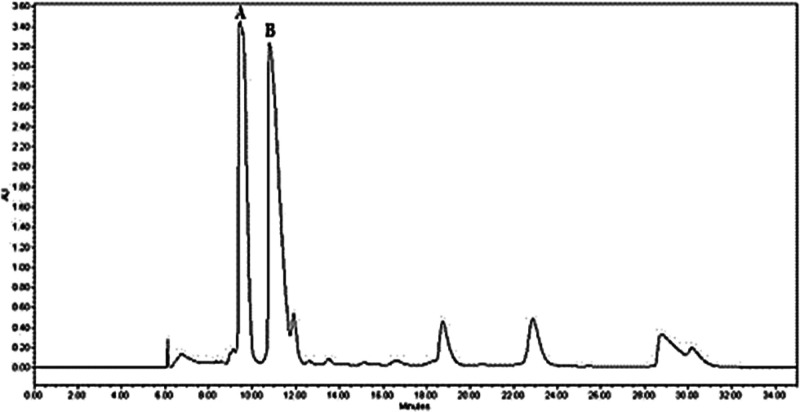
Enantiomeric separation of (±)-2-cicloesil-5-metossi-*2H*-cromene (chromene) via semipreparative HPLC/UV: (A) dextrorotatory stereoisomer; (B) laevorotatory stereoisomer.

Although the enantiomers were successfully resolved, they degraded under the analytical conditions due to dimerization. The isolated products were too unstable for biological testing, so the racemate was used instead. This approach was justified as computational modeling predicted both enantiomers would interact with the target similarly.

### 2.3 Pharmacology

The ability of chromene to selectively inhibit DNA pol-β and pol-α was initially tested using fluorescent cell-free assays, able to reveal the enzymes’ gap-filling activity on a given DNA template. The assays were first run using the reference compounds oleanolic acid (OA, 50 μM), a non-nucleoside DNA pol-β inhibitor,^8^ and cytosine-β-D-arabinofuranoside (AraC, 400 μM), a nucleoside analog of deoxycytidine^20^ that, at high concentrations, competes with dCTP and inhibits DNA pol-α. As shown in Figure 9a,b, results confirmed a significant inhibitory activity compared to the reaction mix devoid of the drugs (RM+).

**Figure 9:**
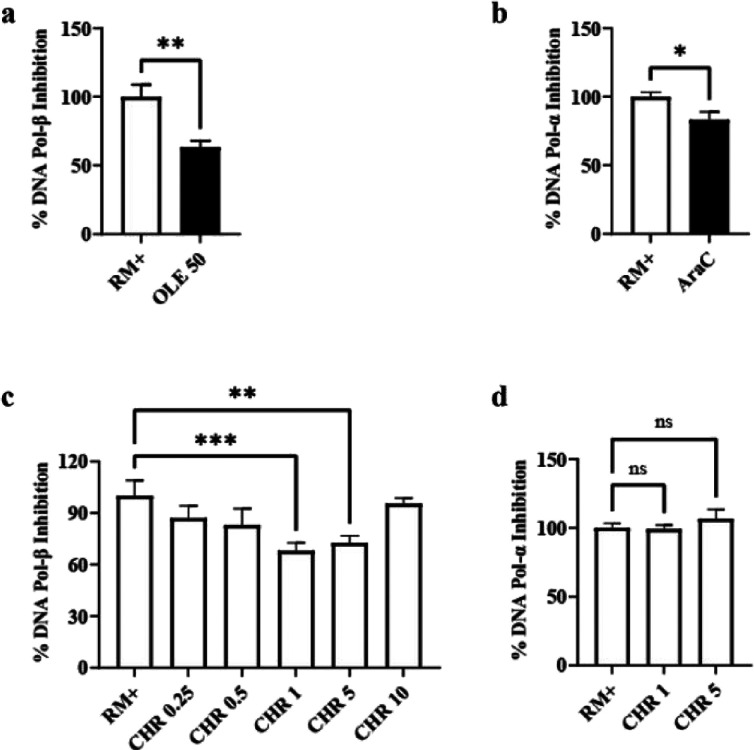
Direct inhibition of either DNA pol-β or primase/DNA pol-α complex in a cell free system. Inhibitory activity was confirmed using reference compounds OA (50 μM) for DNA pol-β (a) and AraC (400 μM) for primase/DNA pol-α (b). RM+ indicates the positive reaction control devoid of any drug. Concentration response curve for DNA pol-β inhibition by chromene (CHR) in the range 0.25–10 μM (c). CHR effects on primase/DNA pol-α inhibition (d). Values are the means ± SEM of 3–6 determinations. *p < 0.05 by Student’s *t* test for significance (a, b) and by one-way ANOVA followed by Dunnett’s multiple comparisons test for significance (c, d); ns = not significant.

A concentration–response curve for DNA pol-β inhibition by chromene was then performed in the range 0.25–10 μM. This range was selected based on previous studies with 5-methoxyflavone, which showed significant efficacy at 10 μM with no increased effect at 30 μM.^8^ As shown in Figure 9c, results with chromene did not prove a linearity between drug concentration and inhibitory effects. In fact, chromene showed a significant but equivalent effect at 1 and 5 μM and was ineffective at 10 μM. Based on these results, the 1 and 5 μM concentrations were further assayed to rule out chromene inhibitory activity on DNA pol-α. Data shown in Figure 9d confirm the lack of effects on pol-α. To assess whether chromene could inhibit DNA pol-β also in a biological system, we carried out the methylmethanesulfonate (MMS) sensitization assay, comparing effects on wild-type and pol-β null mouse fibroblasts. Such test is based on cell sensitization to MMS-induced damage under conditions of DNA pol-β inhibition, due to the lack of DNA repair. As shown in Figure 10a, inhibition of DNA pol-β by reference compound 2’-deoxycytidine (DDC, 80 μM)^8^ significantly amplified MMS toxicity compared to MMS alone in wild type 92TAg fibroblasts, as measured by the MTT viability assay. This effect was absent in pol-β null 88TAg fibroblasts, which lack the functional enzyme (Figure 10b). Chromene was added to both cell lines according to the protocol described in detail in the Section 3. A concentration range between 0.25 and 30 μM of chromene was tested for its effects on MMS toxicity on both wild type and pol-β null cells. In agreement with the cell-free assay results, 1 and 5 μM of chromene significantly amplified MMS toxicity on wild type cells (Figure 10a), but had no effect on 88TAg pol-β null cells (Figure 10b), confirming its on-target mechanism of action. Chromene alone was never toxic at any of the concentrations tested (Figure 10a,b).

**Figure 10:**
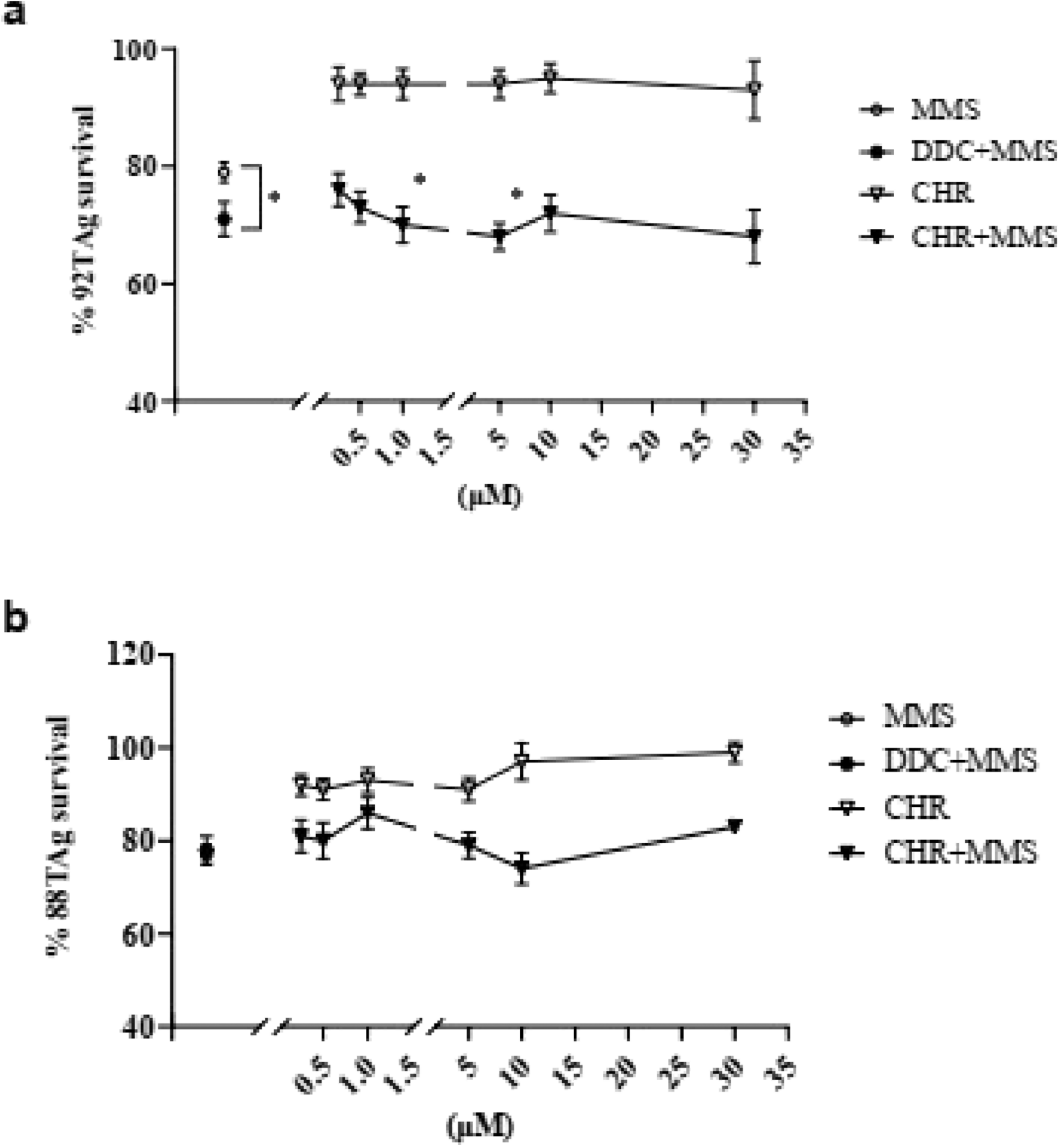
MMS sensitization assay on wild-type 92TAg and DNA pol-β-null 88TAg fibroblasts. Cells were exposed to chromene (CHR) or reference compound DDC (80 μM) for 3 h. MMS was then added for 2 h, after which medium was changed and CHR or DDC alone was added again until the end of the experiment. The control condition with CHR alone was incubated for the same times, but without exposure to MMS. Cell survival at 24 h was analyzed by MTT assay and the resulting concentration–response curves for TAg92 and TAg88 are shown in a and b, respectively. Values are the means ± SEM of 3–8 determinations. *p < 0.05 by one-way ANOVA followed by Dunnett’s multiple comparisons test for significance.

After confirming that chromene acts as a DNA pol-β inhibitor, we proceeded to investigate its potential neuroprotective effects against Aβ-induced neurotoxicity. For the experiments, 1 or 5 μM chromene were added to pure rat cortical neurons for 45 min prior to addition of 100 nM oligomeric Aβ_(1–42)_ and maintained during the entire treatment. Specifically, to assess whether chromene was effective against Aβ-induced neuronal death through the inhibition of DNA pol-β-dependent DNA replication, we analyzed apoptosis and cell-cycle progression via flow cytometry using propidium iodide (PI) staining. Analysis of S phase distribution showed that reduced apoptosis (Figure 11a) was paralleled by inhibition of Aβ-triggered cell-cycle re-entry (Figure 11b) in the presence of both 1 and 5 μM chromene, which did not affect neuronal cell-cycle distribution and survival per se (Figure 11a,b).

**Figure 11:**
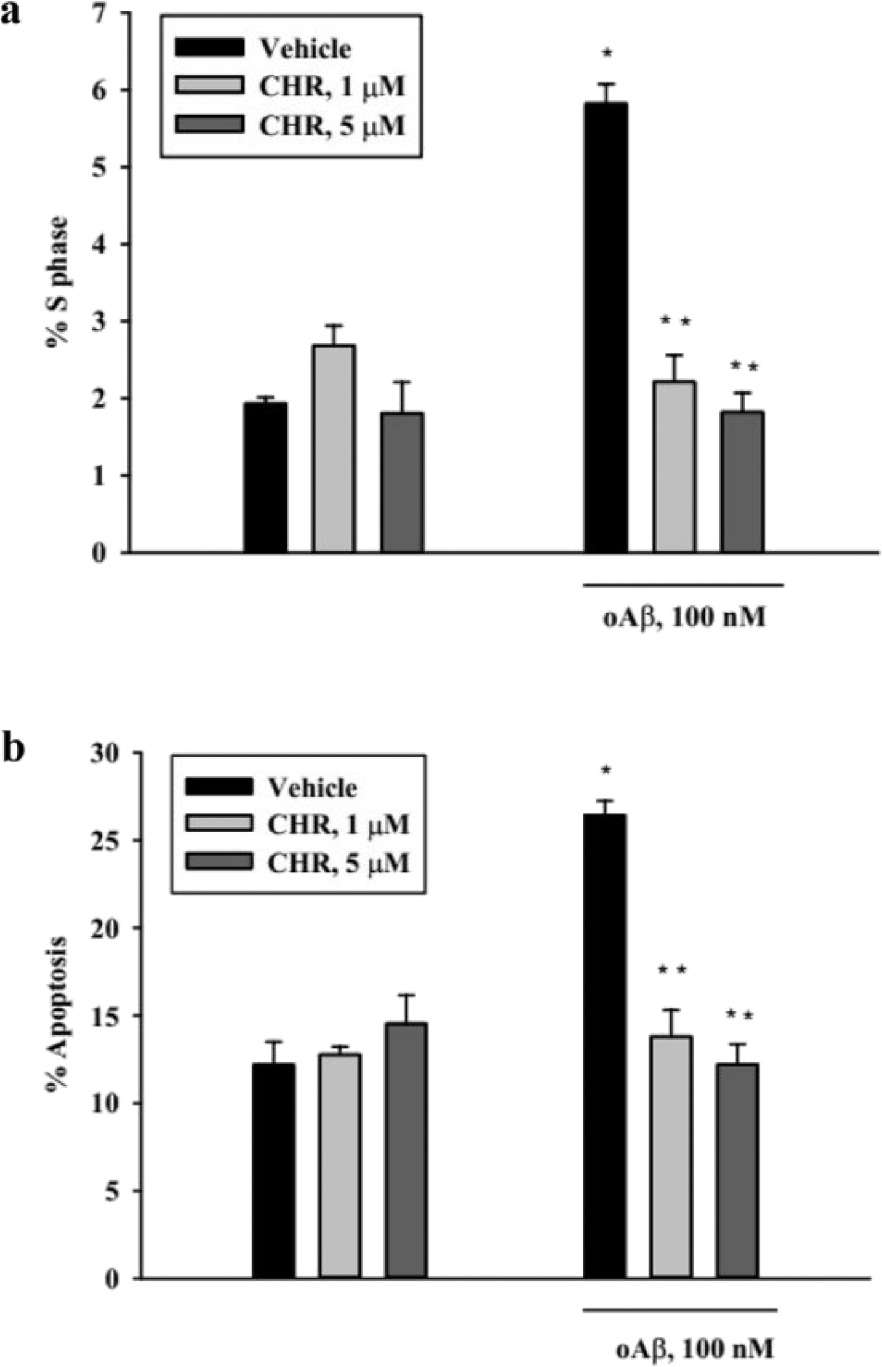
Effects of chromene (CHR) on Aβ-triggered cell-cycle induction (a) and apoptosis (b). Pure neuronal cultures were exposed to 1 or 5 μM CHR for 24 h in the absence or in the presence of 100 nM oligomeric Aβ_(1–42)_ (oAβ). The percentage of neurons in the S phase (a) and the percentage of apoptotic neurons (b) were scored by cytofluorimetric analysis of propidium iodide-labeled samples. Values are means ± SEM of 3–4 determinations. *p < 0.05 oAβ vs control (vehicle); **p < 0.05 vs oAβ alone (one-way ANOVA + Holm Sidak test).

Hence, chromene effectively reduced Aβ-induced apoptosis and suppressed aberrant cell-cycle re-entry in cortical neurons, a hallmark of Aβ-driven neurodegeneration. These findings align with the “cell-cycle hypothesis” of AD, where DNA pol-β-mediated DNA replication in postmitotic neurons contributes to apoptosis.

While 5-methoxyflavone has demonstrated favorable brain uptake in PET studies,^21^ SwissADME prediction carried out for both compounds suggests that chromene may be superior in terms of blood-brain barrier (BBB) permeability and lipophilicity-driven bioavailability. Its lower TPSA (18.46 Å^2^ vs 39.44 Å^2^) and higher fraction of sp^3^ carbons (0.50 vs 0.06), may favor passive diffusion across the BBB. While both compounds are predicted to have high gastrointestinal absorption, chromene lower aromatic heavy atom count (6 vs 16) and reduced CYP3A4 inhibition (avoiding potential drug–drug interactions) further enhance its drug-like profile.

Overall, our development of chromene represents a significant advancement over the parent compound 5-methoxyflavone, demonstrating improved selectivity for DNA pol-β while retaining potent neuroprotective effects against Aβ-induced apoptosis and cell-cycle dysfunction.

Molecular modeling, synthetic optimization, and functional assays collectively validate chromene as a promising therapeutic candidate against Aβ-induced neurotoxicity, although further refinement — particularly regarding enantiomer stability and concentration-dependent efficacy— is warranted.

## 3 Materials and Methods

### 3.1 Molecular Modeling

To generate novel scaffold variants of 5-methoxyflavone, we applied a scaffold-hopping approach using Cresset’s Spark (v10.5.5) https://www.cresset-group.com/spark/; last accessed February 2024. The computational workflow involved (1) scaffold generation through systematic structural modifications and (2) evaluation using field-based similarity metrics, including overall complementarity (integrating steric and electrostatic components), electrostatic alignment (complementarity R), and shape matching (complementarity Rho). The resulting scaffolds (cmd1–cmd16) were ranked based on these metrics to identify optimal candidates. Molecular modeling studies and visualization were conducted using Flare V10.01 (Cresset, Cambridgeshire, UK).^11,12^ The X-ray crystal structures of human DNA pol-β (PDB code: 1BPX; resolution: 2.40 Å) and DNA primase (PDB code: 4RR2; resolution: 2.65 Å) were obtained from the Protein Data Bank (PDB). The retrieved protein structures were carefully inspected and refined to ensure a suitable starting conformation.

Missing hydrogen atoms were added to the structures. Notably, no small-molecule inhibitors have been cocrystallized with these proteins to date. The target binding sites were defined based on our previous work.^8^

Structural optimization was performed using Flare (v 10.0.1) to relax the protein conformations and resolve steric clashes. Protonation states of ionizable residues were assigned to ensure accurate representation of electrostatic interactions during docking. Finally, water molecules and extraneous ligands were removed from the PDB structures prior to further analysis.

(*S*)-chromene and (*R*)-chromene were generated and minimized using Flare v10.01. The docking calculations were performed in default mode, utilizing the “Very Accurate but Slow” method. The binding modes of the enantiomeric compounds were individually evaluated (*in silico* deracemization) by analyzing their highest-scoring docking poses.

### 3.2 General Information on Synthetic Procedures

All reactions were carried out under magnetic stirring refluxed under nitrogen atmosphere unless stated otherwise. All starting materials, reagents and solvents were obtained from Sigma-Aldrich (Milan, Italy) and were used without further purification. All reactions were monitored by thin-layer chromatography (TLC) on silica-plated aluminum sheets (Silica gel 60 F254).

The ^1^H NMR spectra were measured at 25 °C with a JEOL ECZR 600 MHz (JEOL Europe B.V. Nieuw-Vennep, The Netherlands, CDCl_3_). Chemical shifts are reported in ppm downfield from tetramethylsilane. 1D NOE experiments were carried out using the JEOL sequence “noe_1d”.

Chiral separation of chromene was performed by HPLC-UV analysis with an instrument consisting of a Waters 1525EF binary HPLC pump, a Waters 2996 diode array detector and a Waters 717 plus autosampler (Waters Corporation, Milford, MA, USA).

The optical activity was measured on dichloromethane solutions of the two enantiomers by an ATAGO automatic polarimeter, model AP-300 (ATAGO CO., LTD, Tokyo, Japan) at a temperature of 20 °C and with 589 nm light (sodium D line).

### 3.3 Synthetic Procedures

#### 3.3.1 Synthesis of 2-Methoxy-6-hydroxyacetophenone

One g of 2,6-dihydroxyacetophenone was dissolved in 10 mL of anhydrous acetone (previously distilled over KMnO_4_ and stored over anhydrous CaSO_4_). 909 mg of anhydrous K_2_CO_3_ (previously dried in an oven at 120 °C) and, after a few minutes, 624 μL of Me_2_SO_4_ were then added. The reaction was left at 50 °C for 2 h. The course of the reaction is monitored by TLC (petrol ether/ethyl acetate 8:2, *R*_f_ 0.43).

Work up: after cooling, the mixture was added with H_2_O, diluted HCl until pH 5, and then extracted with CH_2_Cl_2_, anhydrified and evaporated. Purification by silica gel chromatography (petrol ether/ethyl acetate 9:1) provide the desired product. Yield = 98%.

^1^H NMR (600 MHz, CDCl_3_) δ 13.26 (*s*, OH), 7.34 (*t*, H-4), 6.57 (*d*, H-3), 6.39 (*d*, H-5), 3.89 (*s*, OCH_3_), 2.67 (*s*, CH_3_).

#### 3.3.2 Synthesis of 2-Cyclohexyl-5-methoxychroman-4-one

To a solution of 1 g 2-hydroxy-6-methoxyacetophenone in 20 mL MeOH, cyclohexanecarbaldehyde (668 μL) and pyrrolidine (124 μL) were added. The reaction was left overnight at 50 °C. The course of the reaction is monitored by TLC (petrol ether/ethyl acetate 7:3, *R*_f_ 0.35).

Work up: after cooling, 100 mL of EtOAc were added to the mixture, which was then washed with 0.5 M aqueous NaOH (100 mL) followed by 0.5 M HCl (100 mL) and aqueous sodium chloride (20 mL). The resulting organic solution was dried over sodium sulfate, filtered, and concentrated to a residue. Purification by silica gel chromatography (petrolether/ethyl acetate 8:2) provide the desired product. Yield = 37%.

^1^H NMR (600 MHz, CDCl_3_) δ 7.35 (*t*, H-7), 6.56 (*d*, H-6), 6.48 (*d*, H-8), 4.11 (*m*, H-2), 3.90 (*s*, OCH_3_), 2,70 (*dd*, H-3a), 2.61 (*dd*, H-3b), 1.80–1.11 (*m*, cyclohexyl).

#### 3.3.3 Synthesis of 2-Cyclohexyl-5-methoxychroman-4-ol

Asymmetric transfer hydrogenation ruthenium-catalyzed:^15^ 1.12 mL (7.49 mmol, 5 equiv) of DBU (1,8-diazabicyclo [5.4.0] undec-7-ene) and 113 μL of formic acid (3.00 mmol) were dissolved in acetonitrile (3.0 mL). The solution was sparged with nitrogen for 15 min, then warmed to 40 °C. Separately, 2-cyclohexyl-5-methoxychroman-4-one (400 mg, 1.50 mmol) and Noyori catalyst (6.50 mg, 0.01 mmol, 0.01 equiv) were dissolved in acetonitrile (2.0 mL). The solution was sparged with nitrogen for 15 min, added to the DBU/formic acid mixture and then left for 24 h. Work up: the mixture was diluted with MTBE (*tert*-butyl methyl ether) (11 mL), washed with 1 M aqueous tartaric acid (11 mL) followed by 1 M aqueous NaHCO3 (11 mL). The organics were dried over MgSO4, filtered, and concentrated. The product of interest is not present in the raw material.

Reduction with NaBH_4_: 400 mg of 2-cyclohexyl-5-methoxychroman-4-one were dissolved in MeOH (20 mL). The solution was cooled to 0 °C, and then 116 mg of NaBH_4_ (3.0 mmol, 2 equiv) were slowly added. The course of the reaction is monitored by TLC (petrol ether/ethyl acetate 7:3, *R*_f_ 0.63 and 0.67). The reaction ended after 4 h.

Work up: water and 3 N HCl is added to bring the pH to 5. After extraction with CH_2_Cl_2_, the organic phase was washed with saturated NaCl solution, dried over sodium sulfate and concentrated to a residue. Purification by silica gel chromatography (petrol ether/ethyl acetate 9:1) provide the desired product. Yield = 90.65%. 1H-NMR (600 MHz, CDCl_3_) δ 7.09 (*dd*, H-7), 6.49 (*d*, H-6), 6.44 (*d*, H-8), 5.12 (*dd*, H-4), 3.89 (*s*, H-Me), 3.75 (*ddd*, H-2), 2.30 (*ddd*, H-3a), 1.92–2.02 (*m*, H-3b), 1.84–1.02 (*m*, cyclohexyl).

#### 3.3.4 Synthesis of (±)-2-Cyclohexyl-5-methoxy-*2H*-chromene (Chromene)

100 mg of 2-cyclohexyl-5-methoxychroman-4-ol were dissolved in DMSO dry (2 mL). The solution was heated at 145–160 °C under reflux condenser. The course of the reaction is monitored by TLC (petrol ether/ethyl acetate 8:2, *R*_f_ 0.58). The reaction ended after 2 h. The reaction was cooled to room temperature under nitrogen and the solvent removed by lyophilization. Yield = 94%.

^1^H NMR (600 MHz, CDCl_3_) δ 6.97 (*t*, H-7), 6.68 (*d*, H-4), 6.36 (*d*, H-6), 6.34 (*d*, H-8), 5.61 (*dd*, H-3), 4.48 (*m*, H-2), 3.75 (*s*, OCH_3_), 1.70–1.06 (*m*, cyclohexyl).

#### 3.3.5 Chiral Resolution of (±)-2-Cyclohexyl-5-methoxy-*2H*-chromene (Chromene)

Chiral resolution of the racemic mixture (chromene) was performed using a semipreparative chiral column (4-methylbenzoate Lux 5 μM cellulose-3, 250 × 10 mm). Elution was carried out in isocratic conditions using hexane and isopropanol in a 9:1 ratio at a flow rate of 2.5 mL/min. Each run separated 250 μL of a solution in hexane (10 mg/mL) of chromene. The UV detector was set at λ = 254 nm (λ_max_ = 280 nm).

### 3.4 Drugs

The β-amyloid 1–42 peptide [(Aβ_(1–42_)] was purchased from Bachem Distribution Services GmbH (Weil am Rhein, Germany) and aggregated into oligomers as previously described.^22^ Briefly, lyophilized Aβ_(1–42)_ was initially suspended in dimethyl sulfoxide (DMSO) to a 5 mM concentration, then diluted to 100 μM in ice-cold Dulbecco’s modified Eagle’s medium-F12 (DMEM-F12; Thermofisher Scientific, Waltham, MA, USA). The suspension was allowed to oligomerize by an overnight incubation at 4 °C. For experiments on primary neuronal cultures, Aβ_(1–42)_ was used at a final concentration of 100 nM, in the presence of the ionotropic glutamate receptor antagonist MK-801 (1 μM; Merck, Darmstadt, Germany) to avoid the potentiation of endogenous glutamate. Control experiments were carried out under identical conditions except for the addition of the peptide. Cytosine-β-D-arabinofuranoside (AraC; Merck) and dideoxycytidine (DDC; Merck) were resuspended in sterile DMEM-F12. Oleanolic acid (OA; Merck) was dissolved in DMSO at a 20 mM concentration. Methylmethanesulfonate (MMS) and [3-(4,5-Dimethylthiazol-2-yl)-2,5-Diphenyltetrazolium Bromide] (MTT) were purchased from Merck.

### 3.5 Cell Lines

Survival experiments were carried out using the mouse embryonic fibroblast 92TAg and 88TAg cell lines from American Type Culture Collection (ATCC, Manassas, VA, USA). The 88TAg line is derived from DNA pol-β null embryos at day 14.5 of gestation and established by transfection with an expression vector for SV40 large T antigen.^23^ The cell line is thus DNA Polymerase beta null [−/−] and additionally transgenic for lambda LIZ (LacI/cII). This matched pair is wild type for DNA pol ι.^24^ Both wild-type and pol-β null cells were maintained at 5% CO_2_ and 37 °C in DMEM supplemented with 10% fetal bovine serum (FBS) and penicillin/streptomycin (Thermofisher). For MTT experiments, cells were plated on 48 well-microplates at a density of 7 × 10^3^/well and serum-deprived at the time of treatment.

### 3.6 Pure Neuronal Cultures

Pure neuronal cortical cultures were obtained from Sprague–Dawley rats (Charles River, Monza, Italy) at embryonic day 15, as previously described.^8^ Briefly, cortices were dissected in a Ca^2+^/Mg^2+^ free buffer, then subjected to low-speed centrifugation and mechanical dissociation in plating medium, consisting of Neural Basal medium supplemented with B27 and 50 units/mL penicillin plus 50 μg/mL streptomycin. Cortical cells were plated at a density of 400 × 10^3^ on 24-well Nunc microplates precoated with 0.1 mg/mL poly-d-lysine. To prevent non-neuronal cell proliferation, after 18 h from plating AraC (10 μM) was added to the medium for the next 72 h. This method yields >99%-pure neuronal cultures. All animal experimental procedures were carried out in accordance with the Directive 2010/63/EU for care and use of experimental animals, and were approved by the Institutional Animal Care and Use Committee of the University of Catania.

### 3.7 Human DNA Pol-β and Pol-α Assays

DNA pol-β and pol-α inhibition were evaluated with selective assay kits (both from Profoldin, Hudson, MA, USA; catalog no. DPB100 KE and HDPA100 KE1, respectively), strictly following the manufacturer’s instructions.^8^ Briefly, chromene was incubated at RT for 30 min in a reaction mix containing a gapped double stranded DNA template and dNTPs, both provided with the kit, in addition to the human DNA pol-β enzyme or pol-α enzyme (Trevigen, Gaithersburg, MD, USA). The reaction was then stopped by addition of a provided buffer containing a fluorescent dye, selectively incorporated by repaired DNA duplexes but not in unrepaired, gapped DNA. Fluorescence was measured at 535 nm with the excitation wavelength at 485 nm on a VarioskanTM Flash Multimode Reader (Thermofisher). Positive (without chromene) and negative (without the enzymes) reaction mix controls were run in parallel. OA (50 μM) was used as a positive control for pol-β inhibition. AraC (400 μM) was used as a positive control for pol-α inhibition. DNA Pol activity was calculated as the ratio between fluorescent signal to no-enzyme (background) signal.

### 3.8 Methylmethanesulfonate (MMS) Sensitization Assay

DNA pol-β inhibition was detected by an assay measuring selective sensitization to the DNA alkylating agent MMS. Amplified toxicity under Pol-β inhibition is present in wild type cells but not in Pol β-null cells tested in parallel.^8^ Briefly, wild type 92TAg and Pol-β knockout 88TAg fibroblasts were preincubated with chromene for 3 h, then pulsed with MMS (500 μM for 92TAg and 250 μM for 88TAg cells) for 2 h. Medium was then changed and chromene added again until the end of the experiment. Cell viability was evaluated at 24 h by the MTT assay.

### 3.9 MTT Assay

After treatment, cells were incubated with MTT (0.5 mg/mL) for 1.5–2 h at 37 °C. Medium was removed and DMSO was added for 15 min at 37 °C for cell lysis. Formazan production by MTT reduction was evaluated by reading at 545 nm in a VarioskanTM Flash Multimode Reader (Thermofisher).^8^

### 3.10 Apoptosis and Cell Cycle Analysis

Neuronal cells were harvested by mild trypsinization and immediately fixed with ice-cold 70% ethanol in PBS. Cells were processed for analysis after a minimum of 24 h of incubation at −20 °C. Briefly, cells were washed with PBS, incubated with RNase (100 μg/mL; Sigma) for 1 h at 37 °C, to remove residual RNA, and incubated with propidium iodide (PI, 50 μg/mL) for 10 min. DNA content and ploidy were assessed using a Beckman-Coulter FC500 flow cytometer to determine the % of apoptotic cells and cell cycle distribution.^8^ Cell-cycle distribution profiles were analyzed with the ModFit LT software program. Apoptotic cells were scored from the area of hypoploid DNA preceding the G0/G1 DNA peak.

### 3.11 Statistical Analysis

All experiments were run at least in triplicate and carried out at least 3 times. Statistical analyses were performed by Student’s *t* test for significance or by one-way ANOVA followed by Dunnet or Holm Sidak posthoc test for significance, as appropriate. GraphPad Prism Software for Windows or SigmaPlot 12.5 software were used for analyses and graphs.

### 3.12 Computational ADME Prediction

The pharmacokinetic properties of chromene and 5-methoxyflavone were predicted using SwissADME (http://www.swissadme.ch).^25^ The tool was employed to assess key parameters such as gastrointestinal absorption, blood-brain barrier permeability and CYP450 interactions.

## Supplementary Material



## Notes

Figure S1: scaffold hopping from 5-methoxyflavone (PDF)
