# Supplementary material for: (±)-2-Cyclohexyl-5-methoxy-2H-chromene, a Synthetic 5‑Methoxyflavone Derivative, Is a Selective DNA Polymerase‑β Inhibitor with Neuroprotective Activity against β‑Amyloid Toxicity
Source: ACS Chem Neurosci. 2025 Dec 10;17(1):173–81. doi: 10.1021/acschemneuro.5c00712 (PMC12784329; doi:10.1021/acschemneuro.5c00712)
Supplement: Supplementary file 1 [file cn5c00712_si_001.pdf]

(±)-2-Cyclohexyl-5-methoxy-2H-chromene, a synthetic 5-methoxyflavone derivative, is a selective DNA polymerase-β inhibitor with neuroprotective activity against β-amyloid toxicity

Salvatore Guccione[1]‡, Sara Merlo[1]‡, Silvia Tagliapietra[2]‡, Matteo Pappalardo[1], Arianna Binello[2], Alessandro Barge[2], Livia Basile[1], Maria Angela Sortino[3], Giancarlo Cravotto[2], Agata Copani[1*].

[1]Department of Drug and Health Sciences, University of Catania, 95125 - Catania, Italy.
[2]Department of Drug Science and Technology, University of Torino, 10125 - Torino, Italy.
[3]Department of Biomedical and Biotechnological Sciences, University of Catania, 95123 – Catania, Italy.

cmd1

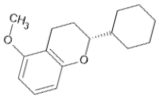

cmd2

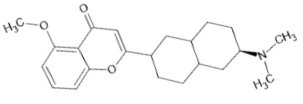

cmd3

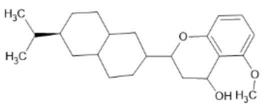

cmd4

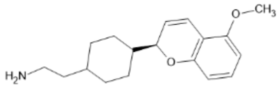

cmd5

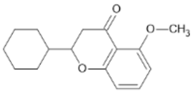

cmd6

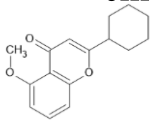

cmd7

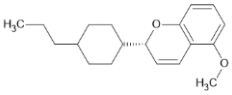

cmd8

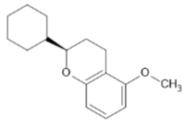

cmd9

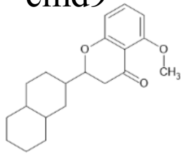

cmd10

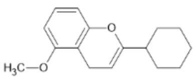

cmd11

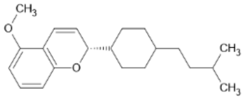

cmd12

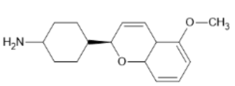

cmd13

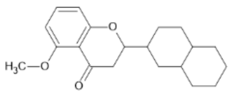

cmd14

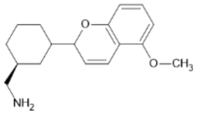

cmd15

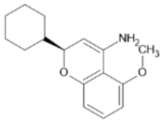

cmd16

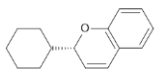

Compound	Complementarity	Complementarity r	Complementarity rho
**cmd1**	0,471	0,571	0,597
cmd2	0,418	0,354	0,433
cmd3	0,435	0,351	0,441
cmd4	0,412	0,299	0,448
cmd5	0,424	0,389	0,51
cmd6	0,416	0,329	0,541
cmd7	0,44	0,044	0,514
cmd8	0,431	0,526	0,496
cmd9	0,441	0,378	0,502
**cmd10**	0,473	0,569	0,597
cmd11	0,42	0,035	0,433
cmd12	0,414	0,472	0,576
cmd13	0,39	0,387	0,539
cmd14	0,41	0,205	0,544
cmd15	0,454	0,456	0,54
cmd16	0,427	0,423	0,369

Supplementary Figure 1. Scaffold hopping from 5-methoxyflavone. Computational design yielded derivatives with evaluated complementarity, complementarity *r* and complementarity *rho* scores. Bold highlights the top-performing derivatives. Structural formulas and scores are displayed.
